# Salivary gland tumors: A 20-year review of clinical diagnostic accuracy at a single center

**DOI:** 10.3892/ol.2013.1750

**Published:** 2013-12-10

**Authors:** WEI-HAN LEE, TE-MING TSENG, HSIN-TE HSU, FEI-PENG LEE, SHIH-HAN HUNG, PO-YUEH CHEN

**Affiliations:** 1 Department of Otolaryngology, Head and Neck Surgery, Taipei Medical University-Shuang Ho Hospital, Taipei 110, Taiwan, R.O.C.; 2 Department of Otolaryngology, Taipei Medical University Hospital, Taipei 110, Taiwan, R.O.C.; 3 Department of Otolaryngology, School of Medicine, Taipei Medical University, Taipei 235, Taiwan, R.O.C.

**Keywords:** salivary gland neoplasm, diagnostic accuracy, malignancy

## Abstract

Obtaining reliable pre-operative diagnosis is crucial in planning treatment for patients with salivary gland tumors. The purpose of this study was to evaluate the accuracy of pre-operative clinical diagnosis of salivary gland tumors managed at a single tertiary university hospital over a period of 20 years. A retrospective analysis of the period between 1992 and 2011 was carried out to review the cases of patients with salivary gland tumors. A total of 101 patients were enrolled and general data were described. Clinical diagnosis was compared with the final pathological diagnosis to reveal the clinical diagnostic accuracy. Of the parotid and submandibular gland tumors, 86 and 67% were benign, respectively. The clinical diagnostic accuracies for diagnosis of parotid tumors as benign or malignant were 100 and 57%, respectively. The clinical diagnostic accuracies for diagnosis of submandibular tumors as benign or malignant were 67%. Therefore, the overall clinical judgment of benign and malignant tumors in the submandibular gland is unreliable. The accuracy for a parotid tumor to be clinically interpreted as benign was 100%. While it is difficult to draw any conclusion for non-parotid gland tumors, surgical intervention should be recommended in patients with parotid tumors clinically suspected to be malignant, and all submandibular, sublingual and minor salivary gland tumors.

## Introduction

The most common type of salivary gland tumor is a slow-growing benign tumor of the parotid gland. The most commonly observed clinical presentation is the appearance of a symptomless mass or lump over the affected area. Less often, signs include fluid draining from the ear, pain, numbness, weakness and trouble swallowing. However, lump formation or swelling of the salivary glands can also originate from non-neoplastic causes, including infection, sialolithiasis, sarcoidosis, amyloidosis and Sjogren syndrome. Salivary gland cancers are rare, comprising only 2% of head and neck tumors. According to the latest data released by the Taiwan Bureau of Health Promotion, the incidence of salivary gland neoplasia is ~1.18 cases per 100,000 individuals in Taiwan. In 2009, 55 mortalities (0.32 per 100,000 males and 0.16 per 100,000 females) associated with salivary gland tumors occurred (1).

As with the majority of benign tumors, benign salivary gland tumors largely appear to be symptomless. However, the most common symptom of major salivary gland cancer is also a painless lump in the affected gland. Although occasionally accompanied by paralysis of the facial nerve, malignancy is hard to determine on the basis of this particular symptom, which only appears in one-third of patients with parotid gland malignancies (2,3). Various attempts have been made to improve the diagnostic accuracy of salivary gland tumors before further measures, including surgery, are necessary. Fine needle aspiration (FNA) cytology appears to be a useful tool, with overall accuracy reported at ~90% (4–12). The majority of previous studies have focused on the comparison of cytological diagnosis and histopathological diagnosis. However, in clinical practice, decisions concerning salivary gland tumor management are not usually based on single examinations but also incorporate information gathered from patient histories, clinical symptoms and signs, physical examinations and imaging studies.

In this study, general data from patients with salivary gland tumors diagnosed and managed at Taipei Medical University Hospital (Taipei, Taiwan) between 1992 and 2011 are presented. The overall impressions of the clinicians prior to surgery were compared with the final pathology results. The main goal of this study was to evaluate, when analyzing patients with salivary gland tumors, the accuracy of pre-operative clinical diagnosis based on various available clinical examinations.

## Materials and methods

The study was approved by the Institutional Review Board of Taipei Medical University Hospital. A retrospective chart review was conducted and data from patients diagnosed with salivary gland tumors at Taipei Medical University Hospital, between 1992 and 2011, were retrieved. Patients who did not undergo surgery or with inconclusive pathology results were excluded. A total of 101 patients were enrolled. Special emphasis was placed on comparing the clinical diagnostic accuracy between various locations of the salivary gland tumor.

### Clinical and pathological diagnosis

Following the exclusion of patients without a complete pre-operative study, including FNA and image evaluation, 53 patients were enrolled in this comparison. Malignancy was suspected when the following observations were made: i) On physical examination the tumor appears immobile or without a clearly defined border; ii) there are signs of facial or other cranial nerve involvement; and iii) there is lymphadenopathy associated with the tumor. Final clinical diagnosis is based on patient history, symptoms/signs, tumor texture under physical examination, imaging studies and FNA.

All 32 patients treated prior to 2008 received computed tomography (CT) scans for their imaging evaluations. From 2008 onwards, 12 of the remaining 21 patients received CT scans and 9 received magnetic resonance imaging (MRI) scans for their imaging evaluations.

Since FNA is not always correct and reliable, it was only considered as a supplement in achieving clinical diagnosis. Among these 53 patients, 26 received FNA. Of these 26 aspirations, 10 were of poor quality. The remaining 16 FNA samples showed non-specific changes. None of the patients received a second FNA.

Pathological diagnoses were purely based on post-operative findings. Correlation between the clinical diagnosis and the pathological diagnosis was taken to indicate an accurate clinical diagnosis.

### Clinical diagnostic accuracy

Clinical diagnostic accuracy was defined as the number of correct clinical predictions of malignancy divided by the total enrolled case numbers. The pathological diagnoses were regarded as the diagnostic standard.

## Results

### General patient data

**Figure 1:**
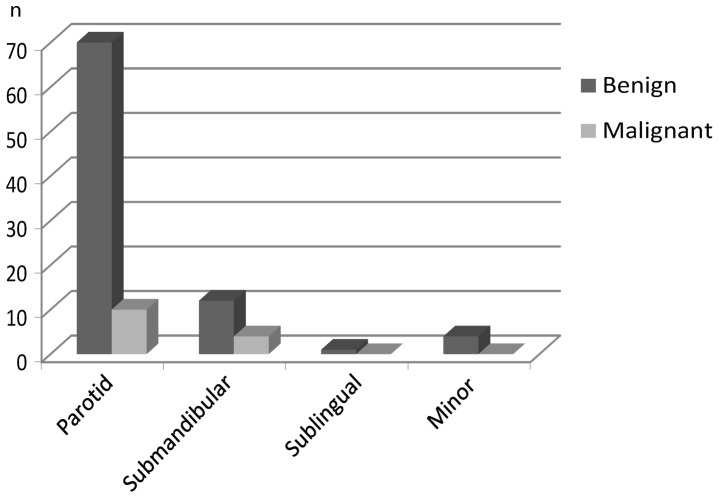
Benign and malignant tumors of the salivary gland grouped according to site.

**Figure 2:**
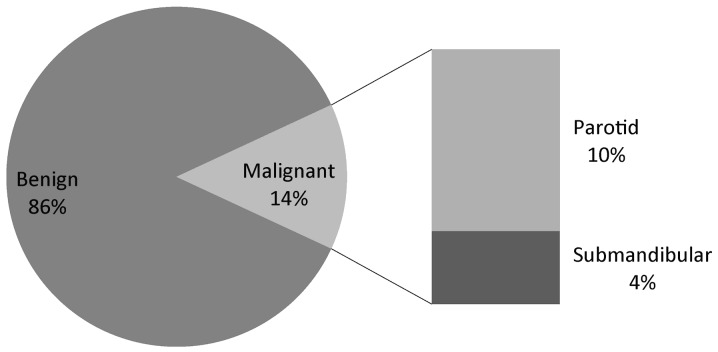
Salivary gland malignancy site distributions, indicating 71% (n=10) of malignancies originated from the parotid gland. The remaining malignancies originated from the submandibular gland.

**Figure 3:**
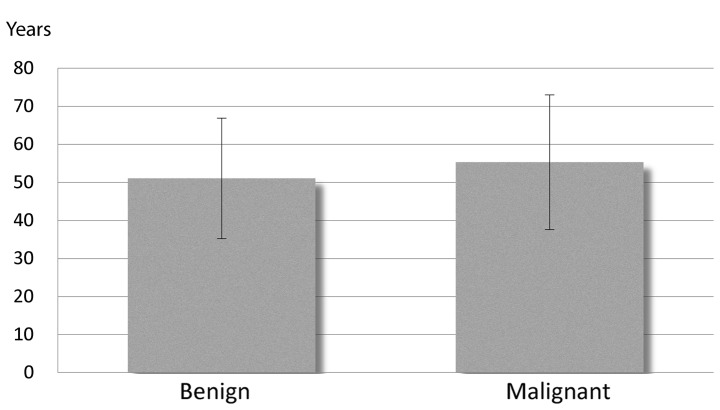
Age distributions for benign and malignant tumors of the salivary gland. No statistical differences were found between the two groups.

Of the 101 patients enrolled, 86% (n=87) had benign pathology. The remaining 14% (n=14) appeared to be malignant in nature. When viewed according to tumor site, the malignancy rates for parotid and submandibular glands were 14 and 33%, respectively. All sublingual and minor salivary gland tumors appeared benign (Figs. 1 and 2). The most common benign histology was mixed tumor (n=63; 72%), followed by Warthin’s tumor (n=23; 26%) and monomorphic adenoma (n=1; 1%) (Table I). Of the 14 patients with malignant pathology, the most common malignant histology was acinic cell carcinoma (n=4; 29%), followed by mucoepidermoid carcinoma (n=3; 21%), adenoid cystic carcinoma (n=3; 21%), carcinoma ex pleomorphic adenoma (n=3; 21%) and epithelial-myoepithelial carcinoma (n=1; 7%) (Table II). The male-to-female ratio for patients with malignant tumors was 6:8. Additionally, no statistically significant difference in mean age was identified between benign and malignant tumor patients (Fig. 3).

### Clinical diagnostic accuracy

The clinical diagnostic accuracies for diagnosis of parotid tumors as benign or malignant were 100 and 57%, respectively. The clinical diagnostic accuracies for diagnosis of submandibular tumors as benign or malignant were both 67% (Table III). There were no cases of malignancy arising from the sublingual gland and the minor salivary gland. Tumors arising from these regions were all correctly interpreted as benign in nature.

## Discussion

Pre-operative diagnosis remains a challenge for clinical otolaryngologists and head and neck surgeons. The present study is the first to discuss this issue from the aspect of overall clinical judgment and diagnosis. The management of benign and malignant tumors in the salivary gland is different, and correct identification of the nature of the tumor can lead to completely different management recommendations. For instance, if a parotid tumor is considered to be at risk of malignancy, a parotidectomy is considered for the majority of cases. Currently, surgical excision or parotidectomy are also standard procedures for benign parotid tumors, with the exception of Warthin’s tumor in the elderly. However, according to a previous study by Eng *et al*, the incidence of temporary facial palsy following parotidectomy is 56–57%, and the incidence of permanent facial palsy is 2–7% (13). In addition, the risk of complication of parotidectomy greatly depends on the experience of the surgeon. At such considerable risk, patients may alternatively choose to monitor the tumor if the chance of malignancy is relatively low. The significant morbidity of facial nerve palsy must be carefully weighed against any possible oncological benefit (14).

Another important issue is the management of the neck lymph nodes. Neck dissection is clearly unnecessary in benign disease. However, if malignancy is suspected, neck dissection is occasionally considered in pre-operative planning. A number of factors, including advanced tumor stages and extracapsular invasions, are considered to determine whether neck dissection is necessary, but malignancy remains the fundamental factor when considering neck dissection (15–18).

The two most common non-surgical strategies for pre-operative diagnosis of salivary gland tumors are FNA cytology and imaging studies. Various studies have concluded that the sensitivity of cytological diagnosis of malignancy ranges between 53 and 90% with positive and negative predictive values of ~90% (4–6,8,10,19–23). However, in studies by Goncalves *et al* and Murai *et al*, the sensitivity of FNA cytology for malignancy was found to be only 42.5 and 42.9%, respectively (10,24). Even with the incorporation of immunohistochemistry, sensitivity did not improve significantly (9). Numerous surgeons question the necessity of FNA cytology and state that results from this procedure rarely define the management of parotid masses, specifically surgical excision. Despite these limitations, FNA cytology remains a technique considered to offer additional information in salivary gland tumor diagnosis. However, even if the cytological results are negative, the test does not replace the need for clinical judgment in the management of a suspected salivary gland neoplasm. At present, the majority of otolaryngologists conclude that no single investigative modality is suitable for diagnosis of specific lesions of the salivary gland (25,26).

Another frequently used diagnostic tool is imaging evaluation. Frequently used imaging techniques include sialography and scintigraphy, plain radiographs, sonography, CT and MRI (27–33). In a study by Klein *et al*, exact diagnosis was only possible in 57% of cases with malignant tumors using sonography (34). Arab *et al* reported that MRI was 73–91% accurate in differentiating between benign and malignant tumors (31). Similar results were demonstrated in a study by Prades *et al*, which reported the diagnostic accuracy of MRI at 83%. The findings of these studies imply that the MRI seems to have reached its limitation in this application (35). In a study by Kan *et al*, positron emission tomography (PET) was evaluated for the differential diagnosis of malignant head and neck tumors from benign lesions. Although 65–70% of benign lesions were correctly identified, there were no patients with salivary gland malignancies enrolled in this study (36). A study by Jeong *et al* revealed that PET/CT provides more accurate diagnostic information for the evaluation of high-grade salivary cancer compared with CT (37). The diagnostic accuracy reported by Jeong *et al* was 97.6%. However, only high-grade salivary cancers were included in the study.

In the present study, not all patients underwent the same imaging procedures. It was not until 2008 that high resolution MRI became available for diagnosis at Taipei Medical University Hospital, and ~50% of the cases treated after 2008 received MRI. This may potentially result in bias toward the clinical diagnosis accuracy as the MRI was generally regarded to be more accurate than the CT scan. However, due to the limited case numbers using MRI (9 cases), it is difficult to draw a conclusion that favors the use of MRI.

Few studies have focused on diagnostic accuracy for salivary gland tumors based on non-surgical or non-invasive procedures. Ultrasound is frequently used together with FNA, and is an effective guidance tool to facilitate the aspiration procedures (6). The first published study discussing the combination of multiple strategies to obtain higher diagnostic accuracy for salivary gland lesions, was carried out by Taylor *et al* in 2011 (5). The authors concluded that sonography, sialography and FNA are effective diagnostic tools guiding the decision for surgical intervention, with CT, MRI and core biopsies declared as useful adjuncts in diagnosis.

In the present study, the subjective judgment of the clinician was also included in an attempt to combat the notion that no single investigative modality can be utilized to diagnose a specific lesion of the salivary gland. Clinical information, including patient history, family history, symptoms/signs and physical examinations, along with the findings revealed by imaging studies and cytology, is essential in informing clinical judgment and decision-making. Therefore, the current study attempted to compare and analyze the results of clinical and pathological diagnosis to assess the reliability of clinical judgments.

The final clinical judgment made by the clinician to distinguish between benign and malignancy was unreliable in submandibular tumors, with an accuracy of only 67%. The only reliable judgment made by the clinician in this study was associated with benign parotid tumors.

In the current study, all sublingual and minor salivary gland tumors appeared to be benign. Case numbers were also low (n=2). Thus, the clinical diagnostic accuracy for tumors in these areas should be regarded as of no clinical significance.

One of the limitations of this retrospective study is that patients who did not receive surgery were excluded and thus it is possible that that the accuracy of clinical diagnosis could be underestimated as there may be a number of patients who refused surgery despite clinically malignant salivary masses. Another limitation of this study is that although subjective judgment is necessary for clinicians to evaluate the disease, this judgment is based on a number of inconclusive and imprecise parameters and the concept is difficult to communicate with colleagues or students. It is also difficult to generalize from subjective results and the value of this study may be questionable in clinical practice.

Nevertheless, based on the results of the current study it is recommended that, in certain situations, delaying treatment should only be advised in cases with clinically diagnosed benign parotid tumors. Surgical intervention should be recommended, if possible, in patients with parotid tumors clinically suspected to be malignant, and in all submandibular, sublingual and minor salivary gland tumors.

The results from the data presented correlate well with the existing literature, as 86% of the parotid gland tumors and 67% of the submandibular gland tumors appeared benign. Overall clinical judgment distinguishing benign and malignant tumors in the submandibular gland was unreliable. While it is difficult to draw any conclusions for non-parotid gland tumors, surgical intervention should be recommended in patients with parotid tumors clinically suspected to be malignant, and all submandibular, sublingual and minor salivary gland tumors.

## Figures and Tables

**Table I:** Histology types for benign salivary gland tumors.

Histology type	Patients, n	%
Mixed tumor	63	72
Warthin’s tumor	23	26
Monomorphic adenoma	1	1
Total	87	

**Table II:** Histology types for malignant salivary gland tumors.

Histology type	Patients, n	%
Mucoepidermoid carcinoma	3	21
Adenoid cystic carcinoma	3	21
Acinic cell carcinoma	4	29
Carcinoma ex pleomorphic adenoma	3	21
Epithelial-myoepithelial carcinoma	1	7
Total	14	

**Table III:** Clinical diagnostic accuracies for salivary gland tumors.

	Total	Accurate	Inaccurate	Accuracy (%)
Parotid				
Benign	28	28	0	100.00
Malignant	14	8	6	57.14
Submandibular				
Benign	6	4	2	66.67
Malignant	3	2	1	66.67
Others				
Benign	2	2	0	100.00
Malignant	0	0	0	0.00
